# Intracranial hemorrhage secondary to disseminated histoplasmosis in AIDS: an uncommon presentation

**DOI:** 10.1590/0037-8682-0830-2020

**Published:** 2021-03-22

**Authors:** Raquel Silveira Bello Stucchi, Athanase Billis, Fabiano Reis

**Affiliations:** 1 Universidade Estadual de Campinas, Faculdade de Ciências Médicas, Departamento de Clínica Médica, Campinas, SP, Brasil.; 2 Universidade Estadual de Campinas, Faculdade de Ciências Médicas, Departamento de Anatomia Patológica, Campinas, SP, Brasil.; 3 Universidade Estadual de Campinas, Faculdade de Ciências Médicas, Departamento de Radiologia, Campinas, SP, Brasil.

A 46-year-old man was admitted to our emergency department with generalized weakness, dyspnea, and a low fever for the previous three days. During the examination, hypotension, tachycardia, hypothermia, and hypoglycemia were observed, and the patient was hospitalized with presumed sepsis of pulmonary origin. A test for human immunodeficiency virus (HIV) performed two months previously was positive (viral load: 47,946 copies/mL) and his CD4+ count was 44 cells/mL. The patient used antiretroviral therapy irregularly.

**FIGURE 1:**
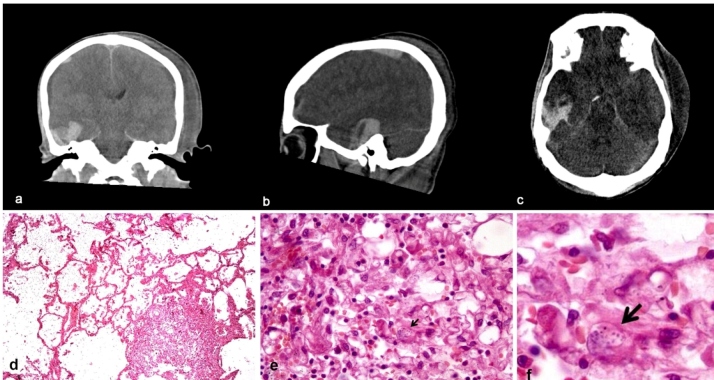
Computed tomography of the skull **(a-c)** showing images suggesting a hyperdense hemorrhagic lesion involving the right temporal lobe and a right acute subdural hematoma with adjacent ipsilateral mass effect. Lung microscopy **(d-f; hematoxylin-eosin)**: the alveolar spaces with macrophage aggregates filled with histoplasma yeast cells (arrows).

Computed tomography (CT) of the abdomen showed hepatosplenomegaly, generalized lymphadenopathy, and ascites. A chest CT revealed consolidation in the right lower lobe and ground-glass opacities involving the basal region. The patient developed acute respiratory failure (that required intubation, mechanical ventilation, and the use of vasoactive drugs) and coagulopathy due to liver and bone marrow failure (pancytopenia). Mydriatic pupils unreactive to light were observed, and brain CT showed subdural and intracerebral hemorrhage in the temporal lobe (Figure 1a-c ). Microscopically, there was focal necrosis in the cortex of the right temporal lobe; this necrosis was wedge-shaped, with a fibrinopurulent exudate containing sparsely distributed histoplasmas. The patient died and necropsy confirmed disseminated histoplasmosis (DH) (Figure 1d-f).

AIDS patients with CD4 counts <150 cells/µL may present with DH^1^. The mortality rate for DH is high among severely immunocompromised patients with AIDS, and the risk factors for death are associated with blood dyscrasia, inflammatory activity, and renal and nutritional impairment^2^. Histoplasmosis should be considered in brain lesions with a granulomatous pattern^3^ as well as in atypical cases with hemorrhagic lesions.
